# Role of ultrasonography in diagnosis of scrotal disorders: a review of 110 cases

**DOI:** 10.2349/biij.5.1.e2

**Published:** 2009-01-01

**Authors:** S Thinyu, M Muttarak

**Affiliations:** 1 Department of Radiology, Lampang Hospital, Lampang, Thailand; 2 Department of Radiology, Chiang Mai University, Chiang Mai, Thailand

**Keywords:** Scrotal abnormalities, ultrasonography

## Abstract

**Objective:**

To determine the role of ultrasonography in diagnosis of scrotal disorders.

**Materials and methods:**

This study was carried out after institutional review board approval was granted, and informed consent was waived. Between January 2005 and January 2007, 144 patients aged 12 years and older with scrotal symptoms, who underwent scrotal ultrasonography (US), were retrospectively reviewed. The clinical presentation, outcome, and US results were analysed. The presentation symptoms were divided into three groups including scrotal pain, painless scrotal mass or swelling, and others. Surgery was performed in 32 patients.

**Results:**

Of 144 patients, 110 had clinical follow-up and constituted the material of this study. The patients ranged in age from 13 to 82 years (mean 38.6 years). Of 110 patients, 84 (76.4%) presented with scrotal pain, 21 (19%) had painless scrotal mass or swelling and 5 (4.5%) had other symptoms. Of the 84 patients with scrotal pain, 52 had infection, 4 had testicular torsion, 7 had testicular trauma, 10 had varicocele, 4 had hydrocele, 1 had epididymal cyst, 1 had scrotal sac and groin metastases, and 5 had unremarkable results. Of the 21 patients who presented with painless scrotal mass or swelling, 18 had extratesticular lesions and 3 had intratesticular lesions. All the extratesticular lesions were benign. Of the 3 intratesticular lesions, one was due to tuberculous epididymo-orchitis, one was non-Hodgkin’s lymphoma, and one was metastasis from liposarcoma. Of the 5 patients who presented with other symptoms, 4 had undescended testes, and 1 had gynaecomastia. US gave incorrect diagnosis in only one patient with scrotal pain.

**Conclusion:**

The most common cause of scrotal pain was infection. The most common cause of scrotal mass or swelling was extratesticular lesion. US plays an important role in the diagnosis of scrotal disorders and in planning for proper management.

## INTRODUCTION

A wide variety of disease processes involving the scrotum may have similar clinical manifestation (eg, pain, swelling or presence of mass). Differentiation of these processes is important for proper management. High-resolution ultrasonography (US) combined with colour Doppler ultrasonography (CDUS) has become the imaging modality of choice for evaluating scrotal diseases [1]. Scrotal abnormalities can be divided into two main complaints, which are scrotal pain and mass. Causes of scrotal pain include inflammation (epididymitis, epididymo-orchitis, abscess), testicular torsion, testicular trauma, and testicular cancer [1-5]. Prompt diagnosis is required to differentiate surgically correctable lesions from abnormalities that can be adequately treated by medical therapy alone. Clinical symptoms and physical examination are often not enough for definite diagnosis due to pain and swelling that limit an accurate palpation of the scrotal contents [2, 4]. For patients presenting with a scrotal mass, it is critical to determine whether the mass is intra- or extratesticular. This is important because the majority of intratesticular lesions are malignant, while extratesticular lesions are usually benign. US is helpful in differentiating extra- from intratesticular lesions [1, 2, 6, 7]. This study was undertaken to determine the role of US in the diagnosis of scrotal disorders in adolescent and adult patients.

## MATERIALS AND METHODS

Institutional review board approval was granted, and informed consent was waived. Between January 2005 and January 2007, 144 patients aged 12 years and older with scrotal symptoms underwent US at Maharaj Nakorn Chiang Mai Hospital. The presenting symptoms were divided into three main groups including scrotal pain, painless scrotal mass or swelling, and others such as undescended testes. Medical records of these patients were reviewed to determine the age, presenting symptoms, US results, treatment and pathological results. Gray-scale and colour Doppler US were performed on all the patients using either high-resolution US units 3000HDI or 5000 HDI (Advanced Technology Laboratories, Bothell, Washington) with a 10-12 MHz linear transducer.

## RESULTS

**Figure 1:**
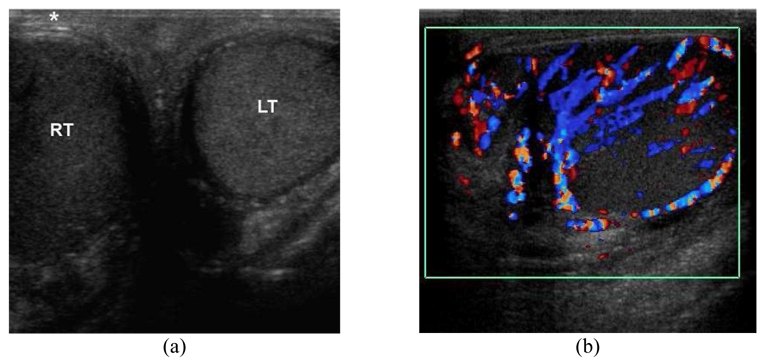
Epididymo-orchitis in a 45-year-old man presenting with painful swelling of the right hemiscrotum for 3 days. (a) Transverse US image of the scrotum shows an enlarged hypoechoic right testis (RT) and a normal left testis (LT). The overlying right scrotal skin is thickened(*). (b) Longitudinal CDUS image of the right hemiscrotum shows increased vascular flow in the right epididymis and testis.

**Figure 2:**
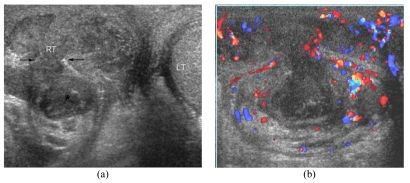
Right necrotising epididymo-orchitis with scrotal wall abscess in a 32-year-old man presenting with painful scrotal swelling and fever for 2 weeks. (a) Transverse US image of the scrotum shows an enlarged heterogeneously hypoechoic right testis (RT) and an heterogeneously hypoechoic tract (arrows) protruding from the right testicular abscess to form a scrotal wall abscess (*). The right testis has lost its well-defined margin. The normal left testis (LT) is partially seen. (b) CDUS image of the right hemiscrotum shows increased vascular flow surrounding the right testis and scrotal wall abscess.

**Figure 3:**
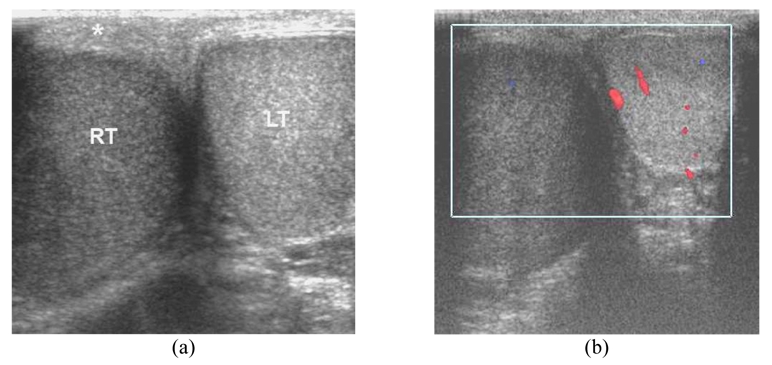
Acute testicular torsion in a 23-year-old man presented with sudden right scrotal pain for 1 hour. (a) Transverse US image shows enlarged, hypoechoic right testis (RT) with thickened scrotal skin (*). (b) CDUS shows no vascularity in the right testis. Note that gray-scale US cannot differentiate between acute testicular tortion and infection. CDUS is helpful to show vascularity in the testis. However, complicated epididymo-orchitis may compromise blood supply.

**Figure 4:**
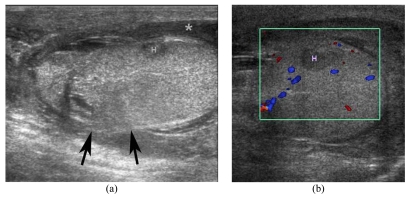
Ruptured testis in a 20-year-old man presenting with a painful swelling of his right hemiscrotum for 1 day after he experienced trauma in the right scrotum while playing football. (a) Longitudinal US shows an indistinct testicular contour (arrows), acute hyperechoic intratesticular haematoma (H) and haematocele (*). (b) CDUS shows no vascularity in the intratesticular haematoma.

**Figure 5:**
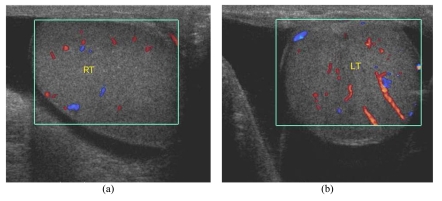
Bilateral hydrocele in a 58-year-old man with history of progressive painless swelling of bilateral hemiscrotum for 2 years. (a&b) Oblique CDUS images show anechoic fluid surrounding bilateral testes, left more than right. There is normal vascular flow in both right (RT) and left (LT) testes.

**Figure 6:**
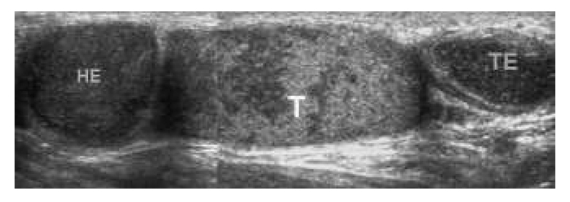
Tuberculous epididymo-orchitis in a 39-year-old man with history of pulmonary tuberculosis, presenting with chronic painless left testicular swelling for 3 years. Composite US images of the left hemiscrotum show nodularly enlarged heterogeneously hypoechoic epidididymal head (HE) and tail (TE), and heterogeneously echoic testis (T).

**Figure 7:**
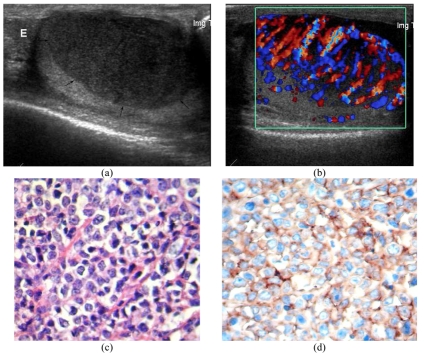
Non-Hodgkin’s lymphoma, diffused large B cell. (a) Longitudinal US image of the right hemiscrotum shows enlarged testis with intratesticular hypoechoic mass (arrows). The right epididymis (E) is normal. (b) CDUS shows marked increased vascularity in the mass. (c) Section of part of testis reveals diffuse infiltration of atypical lymphoid cells that bear large round to oval nuclei, hyperchromatic nuclear chromatin. Prominent nucleoli are noted. These cells individually infiltrate around seminiferous tubules and are densely packed in testicular stroma (H&E stain, x400). (d) The tumour cells are immunoreactive with CD20 (B cell marker staining, x400). (Courtesy of Assistant Professor Charin Ya-in, Department of Pathology, Chiang Mai University, Thailand).

**Figure 8:**
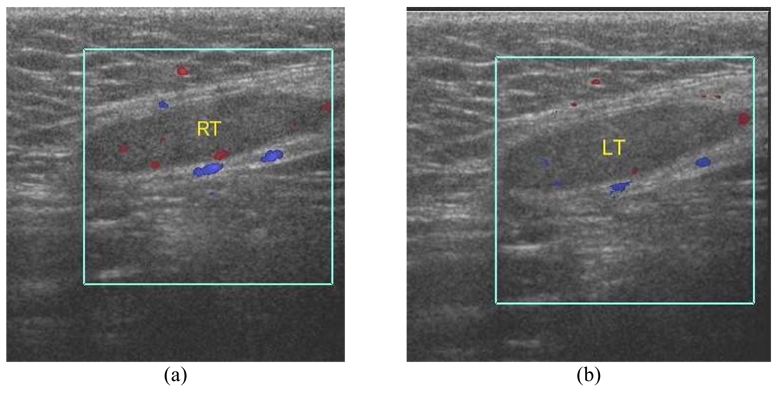
Bilateral undescended testes at inguinal regions in a 19-year-old man with nonpalpable testes in the scrotal sac. (a &b) CDUS images at both inguinal regions show small right (RT) and left (LT) testes.

Of 144 patients, 110 had clinical follow-up and constituted the material of this study. The patients ranged in age from 13 to 82 years (mean 38.6 years). Of 110 patients, 84 (76.4%) presented with scrotal pain (Figs 1-4), 21 (19%) had painless scrotal mass or swelling (Figs 5-7) and 5 (4.5%) had other symptoms (Table 1). Of the 84 patients with scrotal pain, 52 had infection, 27 had non-infection, and 5 had unremarkable results. Of the 21 patients who presented with painless scrotal mass or swelling, 18 had extratesticular lesions and 3 had intratesticular lesions. All the extratesticular lesions were benign. Of the 3 intratesticular lesions, one was due to tuberculous epididymo-orchitis (TBEO), one was non-Hodgkin’s lymphoma (NHL), and one was metastasis from liposarcoma. Of the 5 patients who presented with other symptoms, 4 had undescended testes (Fig 8), and 1 had gynaecomastia, which finally proved to be liver cirrhosis (Table 2).

**Table 1:** Clinical data of 110 patients.

**Presentation symptoms**	**Final diagnosis**	**Number of patients**	**Mean age (years)**
**Scrotal pain (84)**	Infection	52	40.9
	Torsion	4	17.2
	Trauma	7	26.4
	Other eg.varicocele, hydrocele, epididymal cyst, etc.	21	36.2
**Painless scrotal mass or swelling (21)**	Extratesticular	18	40.2
	Intratesticular	3	51.6
**Other symptoms (5)**	Undescended testes	4	27.7
	Gynaecomastia	1	48.0
**Total**		110	38.6

**Table 2:** Causes of scrotal abnormalities.

**Presentation symptoms**	**Final diagnosis**		**Number**
**Scrotal pain (84)**	Infection (52)	Epididymo-orchitis *	29
		Epididymitis	19
		Scrotal sac abscess	3
		Scrotal wall cellulitis	1
	Non-infection (27)	Torsion testis	4
		Testicular trauma	7
		Varicocele	10
		Hydrocele	4
		Epididymal cyst	1
		Metastatic scrotal & groin masses	1
	Unremarkable US finding		5
**Painless scrotal mass or swelling (21)**	Extratesticular(18)	Hydrocele	8
		Epididymal cyst	4
		Varicocele	3
		Seroma in scrotal sac	2
		Inguinal hernia	1
	Intratesticular (3)	TBEO **	1
		Testicular metastases	1
		Non-Hodgkin lymphoma	1
**Others (5)**	Undescended testes (4)	Right inguinal testis	2
		Bilateral inguinal testes	1
		Right anorchia	1
	Gynaecomastia (1)***	Unremarkable US finding	1

* US diagnosis as testicular torsion = 1 case

** TBEO = Tuberculous epididymo-orchitis

*** Finally proved to have cirrhosis as a cuase of gynaecomastia

**Figure 9:**
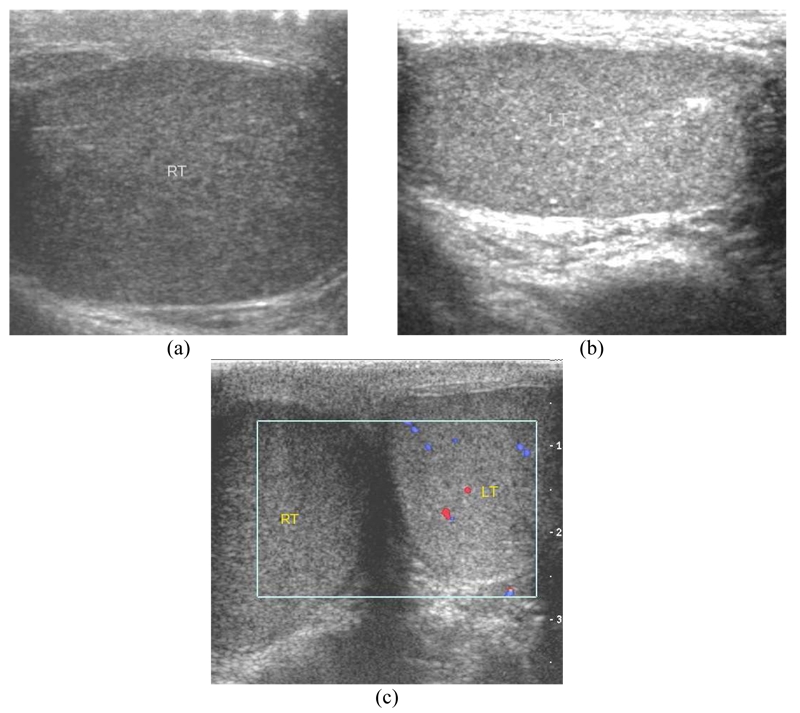
Right epididymo-orchitis with vasculitis in a 24-year-old man with acute right testicular pain for 1 week. (a,b) Longitudinal US images show enlarged heterogeneously hypoechoic right testis (RT) and a normal left testis (LT). (c) Transverse CDUS image of the scrotum shows normal vascular flow of the left testis, but absent vascular flow of the right testis. He was diagnosed with right testicular torsion. Right orchiectomy was performed but pathology turned out to be epididymo-orchitis with vasculitis.

Testicular torsion was found in only 4 patients with a mean age of 17.2 years. Two of these 4 patients presented early, between 1 and 4 hours after the onset of pain. US gave correct diagnosis leading to prompt surgical correction and the testis was salvaged in both patients. The other 2 patients came late, between 4 and 7 days after their symptoms. Orchiectomy was performed after diagnosis of missed torsion. US gave incorrect diagnosis in only one patient with scrotal pain. This patient was a 23-year-old who presented with scrotal pain. US showed mild enlargement of right epididymis and testis with absent vascularity. He was diagnosed to have testicular torsion (Fig 9). Orchiectomy was performed but pathology revealed epididymo-orchitis with vasculitis in the spermatic cord.

In the 7 patients who had history of scrotal trauma, US detected testicular rupture in 4 patients, scrotal haematomas in 2 patients and no abnormality in 1 patient. All 4 patients with ruptured testes underwent operation. Three had partial orchiectomy and one had total orhiectomy.

## DISCUSSION

Common scrotal problems in adolescent and adult male patients that require medical care are scrotal pain and painless scrotal mass or swelling. Bacterial epididymitis or epididymo-orchitis are the most common causes of scrotal pain in adults while torsion is more common in a younger age group [1, 2, 4, 5]. Gray-scale US findings of these lesions, including enlarged epididymis and/or testis with heterogeneous echogenicity, are overlapping but CDUS findings are different. The inflamed epididymis and testis have increased blood flow whereas testicular torsion has decreased blood flow [1-5, 8, 9].

In our study, scrotal pain is more common than painless scrotal mass or swelling. The most common cause of scrotal pain is infection (52/84), which was mostly found in middle-aged men (mean age 40.9 years). Testicular torsion was found in only 4 patients with a mean age of 17.2 years. Two of these 4 patients presented early, between 1 and 4 hours after the onset of pain. US gave correct diagnosis leading to prompt surgical correction and the testes were salvaged in both patients. The other 2 patients came late, between 4 and 7 days after their symptoms appeared. Orchiectomy was performed after diagnosis of missed torsion. The testicular salvage rate is 80% to 100% if surgery is performed within 5 to 6 hours, but the rate decreases to approximately 20% if detorsion is performed after 12 hours following the onset of symptoms [1, 2, 4, 5]. US gave incorrect diagnosis of testicular torsion in one case and surgery was performed but turned out to be epididymo-orchitis with vasculitis in the spermatic cord. This vasculitis may cause testicular ischaemia leading to incorrect diagnosis. However, treatment in this compromised epididymo-orhitis with ischaemia is also surgery.

Scrotal trauma results in contusion, haematoma, fracture or rupture of the testis. Prompt diagnosis of testicular rupture is important because the surgical testicular salvage rate drops from approximately 90% to 45% after 72 hours of onset [1, 2, 4, 5]. This study had 7 patients with scrotal trauma and correct diagnosis was provided, leading to proper management in all.

Other causes of scrotal pain include varicocele, hydrocele, epididymal cyst, and metastases to scrotum and groin in case of supraglottic squamous cell carcinoma. These patients presented with scrotal pain, so they were initially classified in the scrotal pain presenting group, although clinical examination later revealed scrotal mass. They were treated according to their findings. In 5 patients who presented with scrotal pain, and no detectable abnormality on US, this could have been due to prior antibiotic treatment before the US study.

In patients with painless scrotal mass or swelling in this study, extratesticular lesions were much more common than intratesticular lesions. All extratesticular lesions were benign. In the 3 patients with intratesticular lesion, one was TBEO, one was NHL, and one was metastasis from liposarcoma at thigh. There was no primary testicular tumour during the study period. TBEO may present with painless scrotal swelling and can mimic carcinoma. US is helpful in differentiating TBEO from carcinoma. Epididymal enlargement and skin thickening almost always occur in infection but carcinoma rarely involves skin and epididymis [6, 7, 10]. Testicular lymphomas constitute 1-9% of all testicular neoplasms and are the most common testicular neoplasm in men 60 years of age and older [11]. Secondary testicular lymphoma is more common than primary testicular lymphoma. Gray-scale US of testicular lymphoma shows focal or diffuse areas of homogeneous hypoechogenicity which cannot be differentiated from other primary testicular cancers [6]. Testicular metastases are uncommon, and the most common primary sites are prostate carcinoma (35%), lung tumours (19%), colon tumours (9%), and kidneys (7%) [1].

The other presenting symptoms were undescended testes and gynaecomastia. US is the initial imaging modality to detect the location of undescended testes, which most commonly occurs at the inguinal region. The US appearance of undescended testes is similar to one lying within the scrotum, although it is frequently atrophic and small [1, 2]. In this study, undescended testes at the inguinal region could be localised in 3 patients but could not be identified in one patient, who finally proved to be anorchia. US was performed to exclude testicular tumour in one patient with gynaecomastia, which showed no abnormality in the testes. This patient was finally found to have liver cirrchosis, which could be the cause of gynaecomastia.

## CONCLUSION

The most common cause of scrotal pain is infection. The most common cause of scrotal mass or swelling is an extratesticular lesion. US plays an important role in the diagnosis and proper management planning of the scrotal disorders.
